# Supplementary material for: Asynchronous Deep Double Dueling Q-learning for trading-signal execution in limit order book markets
Source: Front Artif Intell. 2023 Sep 25;6:1151003. doi: 10.3389/frai.2023.1151003 (PMC10561243; doi:10.3389/frai.2023.1151003)
Supplement: Supplementary file 1 [file Data_Sheet_1.PDF]

Supplementary Material

1 SUPPLEMENTARY TABLES AND FIGURES

We use the RLlib library Liang et al. (2018) for a reference implementation of the APEX algorithm. Table S1 shows a selection of relevant parameters we used for RL training.

Table S1. Selected RL parameters for APEX algorithm using RLlib Liang et al. (2018) library for training.

Paramter	Value
timesteps_total	300e6
framework	torch
num_gpus	1
num_workers	42
batch_mode	truncate_episode
gamma	.99
lr_schedule	[[0,2e-5], [1e6, 5e-6]]
buffer_size	2e6
learning_starts	5000
train_batch_size	50
rollout_fragment_length	50
target_network_update_freq	5000
n_step	3
prioritized_replay	False

Figure S1 shows confusion matrices interpreting the oracle signal scores as probabilities over the three classes: down, stationary, and up. The predicted class is thus the one with the highest score.

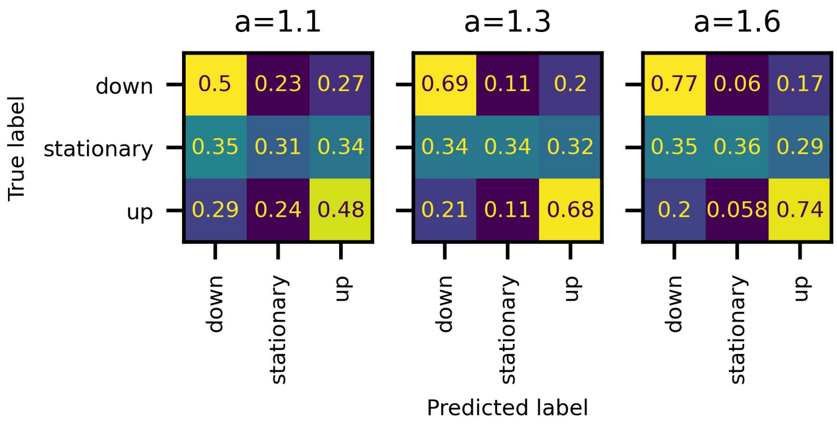

Figure S1: Confusion matrices of the artificial oracle signal for three noise levels, from low to high noise.

REFERENCES

Liang E, Liaw R, Nishihara R, Moritz P, Fox R, Goldberg K, et al. Rllib: Abstractions for distributed reinforcement learning. *International Conference on Machine Learning* (PMLR) (2018), 3053–3062.
